# From Savannas to Settlements: Exploring Cognitive Foundations for the Design of Urban Spaces

**DOI:** 10.3389/fpsyg.2016.01607

**Published:** 2016-10-25

**Authors:** M. Gordon Brown, Charles C. Lee

**Affiliations:** ^1^Space Analytics, WaucondaIL, USA; ^2^Department of Comparative Biomedical Sciences, School of Veterinary Medicine, Louisiana State University, Baton RougeLA, USA

**Keywords:** urban design, embodied space, prospect-refuge theory, grid-form streets, hippocampal cells, system 1 heuristics

## Abstract

Urban configurations in developing countries have become the subject of urban design, with good design promoting economic benefits. Yet, the common practice of urban design lacks a principled scientific foundation. In this respect, cognitive neuroscience could provide a unique perspective and potential foundational insights regarding how embodied cognition links configuration with movement. Although the neural networks that underlie navigation abilities in the brain have been extensively studied, the manner in which these networks might best constrain urban configurations has not been examined specifically. Moreover, it remains an open issue whether the neurological development and functional topographies in the brain that could potentially constrain such urban configurations might also replicate the geometric structures of those environments that were the cradle of human evolution. We propose urban grid-form settlement patterns may be a result of the naturally evolved structures of the human brain. We suggest then that a potential agenda for experimentation and debate could focus on neurological underpinnings of movement choices in urban places. Such an agenda would benefit from bridging a gap between C.P. Snow’s two cultures, i.e., among architects and neuroscientists. Here, we provide a perspective to engender such further dialog on the design of embodied urban spaces and the potential neural systems that may constrain their design.

## Introduction

Cities in developing nations lack well developed urban spatial form and infrastructures upon which they can build, but they have an opportunity to design and create them. “Urban form lasts,” such that designing cities correctly as they grow and develop is of vital importance (OECD, 2015, p. 16). Whether urban population growth will be managed to bring improved social, economic, and aesthetic benefits to new and existing urban populations depends critically on spatial forms that sustain economic and social order. This requires understanding relationships between urban spatial form, human movement and their cognitive underpinnings. However, the received models, metaphors and rules guiding architectural design may not be appropriate nor principled on the basis of those factors most conducive to human perception and cognition.

Vernacular settings and traditional towns are examples of pleasant atmospheres often arising from rather uninteresting units. Such urban atmospheres are most often created by specific materiality, scale, rhythm, color, or formal theme with variations. … In architectural education, we are usually advised to develop our designs from elementary aspects toward larger entities, but our perceptions and experiential judgements seem to advance in the reverse manner, from the entity down to details (Pallasmaa, 2011, p. 8).

Urban design is more than a branch of architecture. In general, architecture aims to control the relationships of entities and details focusing on enclosed and covered spaces, while urban design mainly addresses uncovered and exposed areas, although the purview of these two professions overlap at the intersection of these spaces (Carmona et al., 2010). To whatever extent settings and towns are subject to growth, particularly in the recent decades of commodified real estate, they become a subject of urban design, or per recent critiques, subjected to urban design. Cuthbert (2007) states that urban design has failed to engage with any substantial theory in its cognate disciplines: economics, social and political science, psychology, geography, the humanities. Marshall (2012) adds, “… urban design is at least in part pseudo-scientific … urban design rests on … a foundation of untested hypotheses…”

How then might urban design become more scientifically principled? From the perspective of the cognitive neuroscience, one might suggest that a consideration of the natural evolution of the human brain serves as a likely arena from which to consider the design of urban environments. The question here then might be construed as whether the various navigational networks in the human brain, independently or together, have adaptations suited for particular urban designs? A goal of effective urban design then might be to understand and evoke those perceptual and cognitive processes innate to the evolved circuitry of the brain, as considered below.

## Exploring Neural Constraints on Urban Form

The canonical natural history of human evolution depicts an extensive period of nomadism that transitioned only recently into sedentary enclaves (Brown, 2016). But it took millennia of such sedentism before the construction of authentic cities emerged to support an increasing population (Brown, 2016). City life, with the countless interactions and transactions it supports, likely became possible, perhaps inevitable, when the brain circuitry that had evolved from the nomadic past could eventually, over 50 centuries of trial and error, generate the spaces constituting urban form in the shape of those experienced during that nomadic period (Smith, 2003).

Among the many possible and controversial scenarios for early human societies, human ancestors may have preferred narrow forests along riverbanks providing shelter, food and shade (Cerling et al., 2011) and a long view beyond their immediate locale. Eventually, a transition to open spaces, such as those of savannas, may have occurred (Cerling et al., 2011). The grassy landscape of savannas, stippled or checkered with single or small clusters of trees, likely extended resource possibilities and gave early hominins multiple vistas to find shelter and avoid threats similar in their dimensionality to those paralleling rivers and places.

From this possible scenario, the prospect-refuge hypothesis emerged based on landscape aesthetics (Appleton, 1996), specifically on a human preference for the landscapes of east African savannas. It proposes the human visual system evolved in such open environments to yield information about the utility of spatial-material surroundings important for survival, partly with respect to fight-or-flight conditions in the search for resources (Appleton, 1996; Hildebrand, 1999). Thus, evaluating the visual features of an ‘attractive’ environment include gaging ‘prospect,’ i.e., an unimpeded opportunity to see, and ‘refuge,’ i.e., a place to hide. This assessment guided early humans or human ancestors to perceive and recognize landscape features as they traversed savannas that could be hazards, places of safety as well as resources, friends, foes, potential mates (Appleton, 1996; Hildebrand, 1999). In support of this theory, behavioral studies have generally demonstrated a human preference for prospect and refuge in both exterior and interior environments, as analyzed by Dosen and Ostwald (2013, 2016), which may be supported by specific sensorimotor networks in the brain (Di Dio et al., 2016). Moreover, prospect-refuge theory posits that prospect and refuge are mutually interdependent and much of what is so tacitly attractive about Frank Lloyd Wright’s house designs is said to be their affordance of both prospect and refuge (Hildebrand, 1999). Nevertheless, the possibility and perception of movement is an important element.

Shepard (2001) and colleagues address geometric descriptions and transformations proposing natural selection has shaped inference processes guiding visual perception to reflect properties of the physical world. The functional topographic construction of cortical areas, for instance, likely provides a neural substrate that limits or constrains such perceptual attributes of space. For instance, many prevailing theories of facets of visual perception, such as scale invariance, are based on the network interactions of the topographic maps in visual cortex (Kaas, 1997). The ubiquity of functional topographic arrangements across sensory systems in the brain suggests that the organization of physical properties in the natural world influenced the development of sensory perception (Lee and Winer, 2005; Schreiner and Winer, 2007). Such neural topographic networks thus may indeed be constrained by natural selection or elsewise ontogenetically constrained (Assimacopoulos et al., 2012).

Perhaps more germane to developing a neural theory of urban design is the wealth of research into the navigational networks of the brain. For example, a substantial body of research indicates that the firing of neurons during movement in particular neural structures, e.g., the entorhinal cortical-hippocampal system, can be matched to the locus of movement in the spatial-material world. Neurons in the entorhinal cortex and hippocampus have been shown to be instrumental in cognitive route mapping: place, grid, head direction, and border cells (Solstad et al., 2008). Moser and Moser (2014) provide examples of these navigational neuronal responses:

• Head direction cells fire when mammals face in a certain direction, regardless of the animal’s position.
• Border cells fire when an animal is near its environment’s border, such as a wall or an edge.
• Grid cells fire when an animal passes over equally spaced locations and create a regular, hexagonal grid that looks like the pattern for Chinese checkers.
• Place cells not only receive information about an animal’s surroundings and landmarks, but also continuously update movement—an activity that is actually independent of sensory input.
• Speed cells, provisionally so named and being investigated, react to the speed of an animal’s movement.

Unfortunately, direct tests are lacking concerning whether such topographic or navigational networks contribute principally to the perception or construction of particular urban designs. Nevertheless, it seems reasonable to hypothesize that many of these networks might be best adapted, though shaped by experience, to a particular configuration of spatial elements. In this context, prospect-refuge addresses seeing threats and avoiding being seen by threats, and one could then posit, for example, that head direction neurons might function principally in connection with prospect and border cells with refuge.

Here, the differential activation of navigational networks with respect to prospect and refuge is ripe for testing with the methods currently available to cognitive neuroscience, e.g., fMRI, EEG, behavioral psychophysics, etc. In the laboratory setting, neural activation to photographs or virtual scenes of architectural environments have been used to probe responses to particular design features, e.g., ceiling height, window placement, etc. (Pasqualini et al., 2013; Vartanian et al., 2015; Vecchiato et al., 2015). These studies indicate widespread engagement of not only typical navigational networks, but widespread regions involved in affective and emotive judgements (Vartanian et al., 2015; Vecchiato et al., 2015; Papale et al., 2016).

Of course, probing architectural scenes in the laboratory setting has limitations and the neural activation thus engaged likely does not fully reflect that of the natural environment (Taube et al., 2013). That is, the same advantages that the laboratory setting provides for precisely controlling experimental parameters may also become disadvantageous when probing responses to complex stimuli, like that of a holistic architectural scene (Woods, 1993; Goodwin and Goodwin, 2009; Mitchell, 2012; Taube et al., 2013). Field experimentation is likely to yield more insightful results in this respect (Joye, 2007; Mitchell, 2012). For instance, Chen et al. (2015) used wireless EEG technology to investigate neural activity in different open field environments, i.e., built or wooded regions. Their findings indicate that natural environments promote functional connectivity among brain regions (Chen et al., 2015). Extension of such a field approach using stimuli that are typically simulated in the laboratory environment should provide better insight into those brain networks that underlie prospect and refuge.

More generally though, the neural pathways that have evolved in the human brain may be thought of as generators of experiential judgments that can constrain urban design. The goal for experimental neuroscientists should then be to determine the foundation links between these potential neural networks as adjudicators of the urban spaces that emerge from their natural operations.

## Prospect, Refuge, and Permanent Settlements

Because they predate architectural design by several millennia, Neolithic permanent settlements from about the 8th to the 5th millennia BCE may be the first examples of embodied space (Mellaart, 1965). But it took over 5000 more years before a recognizably urban settlement with fully embodied space was developed. Until about 10,000 years ago, small groups of blood-related *Homo sapiens* lived nomadic lives (Bogucki, 2000, pp. 145–154). With portable materials or those at a site, nomads fashioned dwellings with round shapes grouped in clusters or compounds that afforded refuge. Permanent settlements such as Haçilar and Çatal Huyuk in southeast Asia Minor, dated by archeologists from about the 8th to the 5th millennium BCE, were sustained by pottery and obsidian trading with a mix of some agriculture, herding, hunting, and gathering (Mellaart, 1965). Sedentism enabled accumulating and storing personal property, farming implements, tools and other goods for daily use or trading (Bogucki, 2000, p. 154). Permanent settlements would require protective walls and communal governance would be common with land for farming, pastoral and certain resources functioning as communal property.

**FIGURE 1:**
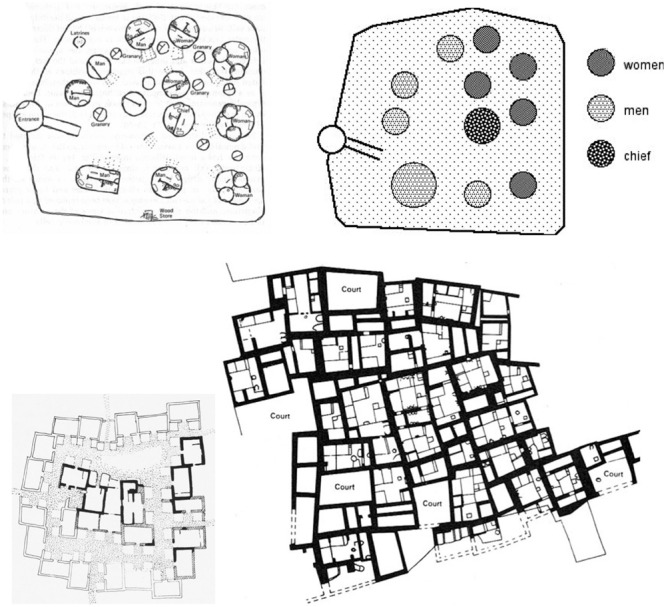
**Design of early settlements.** Above left: Moundang compound (source: Hillier and Hanson, *The Social Logic of Space*); right social/spatial organization. Below left: Hacilar level 6 (source: Mellaart, *Earliest Civilizations of the Near East*, right: part of Łatal Huyuk (source: Haines, *Excavations in the Plain of Antioch).*

As the agricultural revolution deepened its roots over millennia, human populations grew, with more and larger permanent settlements developing. While early sedentist settlements afforded refuge (until they were successfully attacked by more powerful neighbors) prospect within the settlement was missing. Except for the rough, chamfered rectangularity of buildings, Haçilar around the 7th millennium BCE was substantially similar to the cluster form of nomadic settlement layouts: see **Figure 1**. The form of the Haçilar cluster is a simple modification of the form of the Moundang settlement that results from pushing some dwellings against and integral with the wall. Clustered dwellings for several centuries in Haçilar and for several millennia in Çatal Huyuk, with a population of well over 5,000, had rooftop entries, small courtyards but no ground level doorways and no streets. Communal governance and land ownership were common but increased trade made these forms obsolete and a more complex economic and social context made communal decision-making ineffective. This required a fundamental shift in governance, land ownership and spatial form to accommodate movement and visual regulation of strangers (Brown, 2016). It would be many centuries of trial and error before the institutional and spatial structures of these settlements would be able to accommodate the increasing number of transactions that would result. By the fourth millennium BCE, productive alluvial agriculture around the Euphrates stimulated trade, job creation, differences in property and wealth and a profusion of new goods that attracted outsiders (Mumford, 1961, p. 35; Mann, 1986) Uruk and Ur emerged.

With the emergence of large, permanent human settlements like Ur, “… for the first time the stranger was the rule, not the exception, and had to be monitored (Lofland, 1973, p. 9).” A different form of urban configuration that could accommodate both movement and the monitoring of strangers enabling trade, cooperation and security became a necessity. A ruler would be given or would take control of collective property. Ownership of a more productive piece of land might lead to conflict and need to be politically or judicially settled by a ruling power: a king or council (Mann, 1986). These changes required a priestly class to coordinate and run institutions for justice and for trade that would keep transaction costs down (Beaudreau, 2011). The result 5,000 years ago would be a nascent form of the grid with linear urban spaces for prospect (as collective real property) and cellular spaces for refuge (as private real property) arranged orthogonally.

## The Heuristics of Urban Form

Harré (1978, p. 141) proposes that “structure-creating activity is natural to man, that men are architectonic.” In terms of cognitive embodiment, head direction, border and grid cells would help reproduce prospect and refuge as built form. Prospect became collective real property – streets; refuge – buildings abutting prospect became private real property (Brown, 2016). In this regard, could grid-form systems of streets be possible embodiments of human cognition? That many urban grid-form layouts are rectangular instead of hexagonal is a possible example that “endogenous knowledge exhibits autonomous structuration without being reduced to interiorization of exogenous contributions, but rather completing them by reconstructions that go beyond them (Piaget and Inhelder, 1972, p. 804). Aside from this observation that many urban spaces take on grid-like forms, it remains unclear whether similarly organized networks in the brain, e.g., grid cells, directly cause this, although it is tempting to hypothesize such a link because of the nearly universal worldwide patterns of grid-form settlements. It is here that neuroscientists may be able to experimentally probe a purported preference for such grid-form environments.

Historically, it is interesting to note that Greek cities in Asia Minor refined orthogonal patterns into grids. Greek and Roman colonies would be settled on a grid (Castagloni, 1971). Leroi-Gourhan (1993, pp. 336–339) sees grid-form street geometry as a technology that has persisted for millennia. The American Founding Fathers chose the Greek grid for new settlements (Morris, 1888). Particularly remarkable is that the great majority of urban settlements today are orthogonal, mostly grid-form systems of collectively owned streets lined by privately owned buildings. Street grids have longevity because their form yields a very efficient arrangement of prospect and refuge.

Urban grid systems may appear to be composed of uninteresting units, but they’re not formal tessellations. They come in different dimensions, orientations and scales, are easily extended, sometimes interspersed with diagonals and curving boulevards and allow multiple uses over time. The essence of a grid-form street system is mostly orthogonally arranged streets with mostly four-way intersections enabling a combination of route choice and loops of streets for alternate ways of returning to a trip’s starting point. Grids with narrow streets sustain local travel. Grids with multi-lane divided arterial streets, as in Phoenix, enable longer distance movement (Cervero, 2001, pp. 131–132). While grids can have a variety of block sizes and shapes like the City of London, be radial like Amsterdam, or incomplete for topographic and other reasons, they typically have longer and more linearly connected thoroughfares.

Prospect theory (Kahneman and Tversky, 1979) and its relations point out a number of common biases and heuristics limiting rational judgment.^1^ Most judgment and heuristics errors are regarded as instances of using system 1, a collection of multiple fast and unconscious systems operating in parallel. System 2, slow, serial, and conscious, is regarded as rational (Carruthers, 2009; Kahnemann, 2011). But system 1, which developed through the same evolutionary processes that affect how we orientate ourselves to make prospect and refuge choices, is not necessarily irrational. Humans use heuristics effectively, especially when addressing similar decision problems. If a different heuristic were required for every slightly different decision-making environment, we would need an unworkable multitude of heuristics and not be able to generalize to previously unencountered environments (Gigerenzer and Goldstein, 1996). System 1 is the same as, similar to or closely related to embodied cognition because they are both not easily altered and are mostly heuristic. Such system 1 heuristics are what Gigerenzer (2000, p. 18) calls a “marriage of structure with simplicity... in which there is little trade-off between being fast and frugal and being accurate.”

Istanbul’s Grand Bazaar, although it has an orthographic grid, seems an anomaly because so many visitors, as countless travel guides testify, experience it as a maze: La Corbusier was one (Jeanneret, 1965). The Bazaar is not arranged like the Minotaur’s Labyrinth but its pedestrian channels are narrow, its walls are lined with a variety of goods and attractions and, most important, are covered with weakly illuminated vaults. This is architecture, not urban design. Being covered, prospect is not perceptible because the profusion of shops, goods and crowds in these narrow channels results in what information theory calls noise. In architecture, controlled intent is applied in every aspect of form to minimize visual and spatial noise. This is neither appropriate nor workable in urban places, which, like savannas where, we became human, are uncovered and exposed but, unlike savannas, harbor many forms of noise.

**FIGURE 2:**
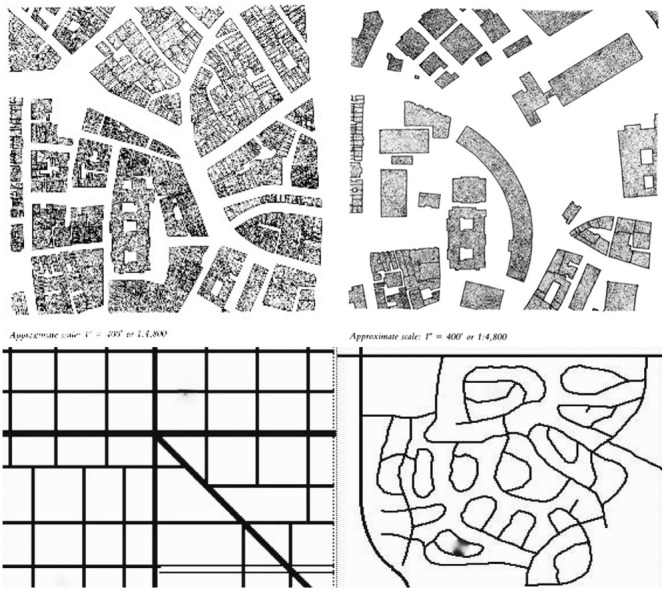
**Examples of urban designs.** Upper left: central Boston 1929. Upper right: central Boston 1980. Source: Jacobs, 1993. Lower left: pre-WWII American grid-form street pattern. Lower left: post-WWII curvilinear-dendritic street pattern. Source: Google Maps.

Following Gibson’s (1979) research on how an environment offers affordances, Proffitt (2006, p. 110) says, “Perceptions are embodied; they relate body and goals to the opportunities and costs of acting in the environment.” As a cognitive embodiment, grid system streets combine structure with simplicity enabling fast, frugal and accurate orientation in any unencountered part of any grid-form environment. Unlike most street patterns consciously configured by designers, grid street systems have long had anonymous authorship. Urban environments making orientation difficult include the curvilinear-dendritic street systems in post-WWII American suburbs. Traditional city grids are exponential networks offering significant choice in movement. **Figure 2** shows both pre- and post-WWII patterns. The upper shows the removal of a roughly orthogonal street pattern to accommodate a major redevelopment. The lower left is a stylized drawing of stitched together grids as in San Francisco and other cities and the right a typical 1970s suburban street pattern development in Denver. Recent American urban planning and design, successfully exported to other cultures, has neglected affording fast, frugal and accurate orientation and produced what can be called disembodied space. At metropolitan levels, these patterns have the form of scale-free networks constituting a regime shift from exponential networks with negative economic consequences (Brown, 2016). We offer as a perspective and hypothesis then that such urban grid-forms may be a result of the naturally evolved structures of the human brain and that an avenue for further research would be to examine whether these neural networks contribute to their emergence.

## Conclusion

Good urban design makes new and redeveloped physical environments spatially and visually attractive and, while prospect and refuge in urban form are necessary, the grid alone is not sufficient. Embodied space includes mystery – partly blocked views, gaps in patterns, elevation changes and what’s around the corner or down at the end (Kaplan and Kaplan, 1989). Many post-WWII urban spatial configurations, because they have split prospect from refuge, fail to afford recognition of hazards, places of safety, resources, opportunities, friends, foes and perhaps even potential mates. Tweaking two-dimensional urban form and linking its uncovered vertical dimensions should add what Ashby (1964) calls requisite variety, the zone between redundancy and noise that enables intelligibility.

As we have suggested above, urban design is ripe for testable hypotheses by both architects and cognitive neuroscientists, addressing issues like optimal street length and width parameters, desirable number of sequences of spatial intervals separating built form elements, how best to incorporate elements of spatial mystery: bent paths, undulations and other level changes, foliage, views and view sheds. Redundancy, uncertainty and informational noise in three dimensions must be considered in cognitive research to be useful in urban design, as should the evolution and structure of human navigational brain networks. As discussed above, although such experimental assessments are generally confined to the laboratory, novel methodologies that combine field experiments and simulations with new technologies, such as wireless EEG caps and wearable tattoo electronics (Chen et al., 2015; Zich et al., 2015), will enable testing new hypotheses addressing the cognitive foundations of spatial embodiment.

## Author Contributions

MB conceived, drafted, and edited this submission. CL reviewed, drafted, and edited this submission. This work was supported in part by a grant from the Louisiana Board of Regents, RCS-RD-A-09, to CCL Parts of this work are based on Brown (2016).

## Conflict of Interest Statement

The authors declare that the research was conducted in the absence of any commercial or financial relationships that could be construed as a potential conflict of interest.

## Footnotes

1 Appleton’s prospect-refuge theory and Kahneman and Tversky’s prospect theory are independent of each other.
